# Short-term changes in the health state of children with group B meningococcal disease: A prospective, national cohort study

**DOI:** 10.1371/journal.pone.0177082

**Published:** 2017-05-18

**Authors:** Iain T. R. Kennedy, Albert J. van Hoek, Sonia Ribeiro, Hannah Christensen, W. John Edmunds, Mary E. Ramsay, Shamez N. Ladhani

**Affiliations:** 1 Immunisation Department, Public Health England, London, England, United Kingdom; 2 Department of Infectious Disease Epidemiology, London School of Hygiene and Tropical Medicine, London, England, United Kingdom; 3 School of Social and Community Medicine, University of Bristol, Bristol, England, United Kingdom; 4 Faculty of Epidemiology & Population Health, London School of Hygiene & Tropical Medicine, London, England, United Kingdom; 5 Paediatric Infectious Diseases Research Group, St Georges University of London, London, England, United Kingdom; Universidade Nova de Lisboa Instituto de Higiene e Medicina Tropical, PORTUGAL

## Abstract

**Objectives:**

The short-term impact of childhood invasive meningococcal disease (IMD) on quality-of-life (QoL) remains largely unquantified. This study aimed to quantify QoL loss at the point when illness was at its worst, and assess health state recovery in the months following illness.

**Methods:**

Parents of children aged <16 years with laboratory-confirmed meningococcal group B (MenB) disease in England, with onset dates from November 2012 to May 2013 were asked to complete a short questionnaire, which included EQ-5DY, a version of EQ-5D for 8–15 year-olds. The parents, or child if able, were asked to complete the questionnaires while considering the child’s health on the worst day of illness and on the date the questionnaires were completed.

**Results:**

The overall response rate was 43% (109/254 children), with no significant differences between respondents and non-respondents. The median time from disease onset to questionnaire completion was 134 days (interquartile range (IQR), 92 to 156 days). After imputation, the median health index was -0.056 (IQR, -0.073 to 0.102) on the worst day of illness, and 1 (IQR 0.866 to 1.000) on the date of questionnaire completion. The respective Visual Analogue Scores (VAS) were 6.5/100.0 (IQR, 0.0 to 20.0) and 95.0/100.0 (IQR, 90.0 to 100.0). The health state of cases with long-term sequelae (n = 41) was significantly worse at follow-up than those who recovered uneventfully (n = 64; 90.0 vs. 98.0; p<0.001), although there was no significant difference on the worst day of illness (5.0 vs. 10.0; p = 0.671).

**Conclusions:**

This work has provided, for the first time, a quantitative estimate of QoL loss at the peak of illness and in the months after MenB disease in children. The magnitude of QoL loss is staggering, with the reported health state being at, or close to, the worst possible outcome imaginable. This study highlights the difficulties in measuring the impact of illness in young children, who often have the highest burden of potentially preventable infectious diseases.

## Introduction

*Neisseria meningitidis* is a major cause of meningitis and septicaemia globally and the single most important infectious cause of childhood deaths in industrialised countries [1]. The meningococci can be distinguished according to their unique polysaccharide capsule into 12 groups, of which four (B, C, W and Y; henceforth MenB, MenC, MenW and MenY) are responsible for most cases of invasive meningococcal disease (IMD) in Europe [1]. In 2014/15, in the United Kingdom, MenB was responsible for 80% of IMD cases in children aged <1 year (101/127 cases), 86% (139/162 cases) in 1–4 year-olds and 75% (49/65 cases) in 5–14 year-olds [2]. MenC is rare because of a successful national immunisation programme against this capsular group, while MenW and MenY usually cause disease in older adults, who often have multiple underlying co-morbidities. However there has been a recent upsurge in Men W cases, prompting changes to the adolescent vaccination schedule [3].

IMD is associated with significant mortality in children and young adults because the illness starts suddenly and progresses rapidly, often before any medical intervention can take place [4]. Of those who survive the infection, high rates of long-term physical and neuro-developmental sequelae have been reported. In a recent, long-term national case-control study, the 245 children with MenB disease diagnosed during 2008–10 had significantly higher rates of bilateral sensorineural hearing loss, disabling amputations, psychological disorders, lower Intelligence Quotient (IQ), and deficits in executive function and multiple aspects of memory compared to 328 age and sex-matched controls [5]. The short-term impact of IMD on quality-of-life, however, remains largely unquantified. This study aimed to quantify the quality of life (QoL) loss at the point when illness was at its worst, and assess health state recovery in the months following illness. This work was conducted as part of Public Health England's (PHE) duty for ongoing surveillance and control of infectious disease, and was reviewed by the PHE Vaccine Preventable Bacterial Infections Forum as well as representatives of the two main meningitis charities (Meningitis Research Foundation and Meningitis UK). The data collected fed into the economic modelling [6], which was considered by the UK’s Joint Committee on Vaccination and Immunisation (JCVI), and subsequently to the introduction of the novel, multi-component MenB vaccine into the national infant immunisation programme in September 2015 [7].

## Material and methods

PHE conducts enhanced surveillance of meningococcal disease in England through the meningococcal reference unit (MRU), which provides a national reference service for confirmation and grouping of invasive meningococcal isolates alongside a free PCR-testing service for patients with suspected IMD [8].

The general practitioners (GP) of children aged <16 years with MRU-confirmed MenB disease with onset dates from November 2012 to end May 2013 were contacted, either by phone or by letter, to inform them of the enhanced surveillance and, if the GP had no objection, a covering letter with a questionnaire was sent to parents. Non-responders received one postal reminder after 4 to 6 weeks. Reminders were not sent after the target of 100 responses had been reached. Children who had died or had insufficient demographic data (name, date of birth, National Health Service number) were excluded.

The questionnaire consisted of three parts. The first was an enhanced surveillance questionnaire which included questions on duration of illness, symptoms at presentation, pre-existing conditions, duration of hospital stay, intensive care admission, and time off from educational activities. We also asked about the presence or absence of long-term sequelae (complications), specifically memory/concentration problems, seizures, hearing loss, amputation (digits or limbs) and “other”, with a free-text box to describe any additional issues. Parts two and three were copies of the 5-dimensional questionnaire for youth developed by EuroQoL (EQ-5DY), which is designed for children aged 8–15 years [9]. The invitation letter asked for the questionnaire to be completed either by the child, if able, or the parent. They were asked to complete two copies of the questionnaire: one while considering the child’s health on the worst day of illness and one representing the date when the questionnaires were completed. Each EQ-5DY consists of two parts. The first is a series of five questions covering the domains of mobility, self-care, daily activities, pain, and emotional wellbeing. Each domain can be scored at one of three levels (no problems, moderate problems, severe problems). The results provide a multi-domain ‘health index’. These can then be transformed using predetermined weightings to provide a single index score. There is no validated weighting for younger age groups; therefore, the standard UK population weights were used. There were very few cases aged >15 years (4/254 eligible cases; 1/109 respondents), therefore, the same questionnaire design was used for all children.

The second part is a single measure of self-assessed or proxy-assessed health state. It uses a ‘thermometer’ or visual analogue scale (VAS) where the parent marks the perceived health state of the child on a line between zero and one hundred, where zero is worst possible health imaginable and 100 is perfect health [10].

**Fig 1:**
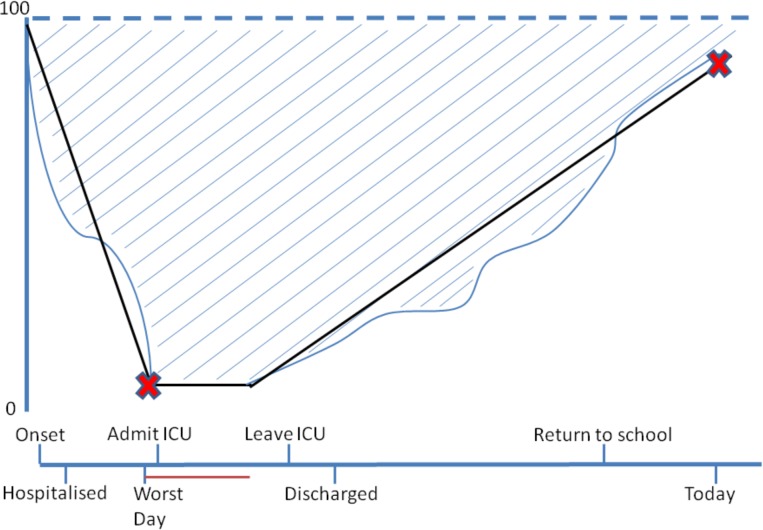
Stylised diagram of possible course of illness and recovery in children with laboratory-confirmed, group B meningococcal disease, with blue lines demonstrating the variable course of illness. Black lines represent the assumptions made about health state lost and regained over time. The red crosses represent points measured in this study. The red line represents the number of days the child was considered ‘most unwell’. The hashed areas represent the QoL lost.

Where missing data meant that the health index could not be completed, results were imputed based on a regression of the health index with the VAS results, a methodology described by Thorrington and colleagues [11]. The quality of life assessment at both time points, along with time parameters (onset date, date of worst health state, date of follow up, number of days at worst health state) were used to produce the health profiles covering time from onset to date of follow up (**Fig 1**). This was done for VAS, health index and health index plus imputed data (**S1 Table**). The standard profile was produced by assuming that changes to health state occurred on a straight-line basis (that is, the change in health state occurs evenly across the each time period). It was further assumed cases started with no QoL loss and that there was no further improvement after follow-up. Cases with pre-existing chronic medical conditions and those missing essential data were excluded.

A simple sensitivity analysis was performed by creating minimum and maximum QoL loss profile scenarios. These were created by changing the assumption that health state changes over time to health state changing instantaneously. The minimum scenario assumes all health loss occurs on worst day of illness, and all health is regained on the first day after the period when the child was in the worst health state. The maximum scenario assumes health loss occurs on the day of onset of illness, and then regained on day of completion of questionnaire. The proportion of health state lost was calculated for each case and then multiplied by the number of days from onset to date of follow-up, to give an estimate of the number of Quality Adjusted Life Days lost during the acute illness.

### Data analysis

Data from the returned questionnaires were entered into a Microsoft Access™ database. Health profiles were generated in Microsoft Excel™. Statistical analysis was performed using R. Wilcoxon rank sum test with continuity correction was used when comparing health states, as EQ-5DY results were not normally distributed. Differences between responders and non-responders were assessed using chi square test or the chi square test for trend, as appropriate.

### Ethical approval

PHE has legal permission, provided by Regulation 3 of The Health Service (Control of Patient Information) Regulations 2002, to process patient confidential information for national surveillance of communicable diseases (http://www.legislation.gov.uk/uksi/2002/1438/regulation/3/made). According to the guidelines of the National Research Ethics Service (www.nres.nhs.uk/EasySiteWeb/GatewayLink.aspx?alId=355), collection of quality of life information from patients comes within the remit of the enhanced surveillance activities of PHE and, therefore, ethics approval was not required. Families of children with IMD were only contacted if their GP raised no objection. The cover letter enclosed with the questionnaire clearly explained the role of PHE, the reason why the family was contacted, what information was being sought and how the information would be used. Completing and returning the questionnaire by post implied consent. All data were anonymised before analysis.

## Results

The overall response rate was 43% (109/254). There was no significant difference in age (p = 0.529) or gender (p = 0.774) between respondents and non-respondents. The median time from onset to completion was 133 days (IQR 91.5 to 156 days). Seventeen children reported having pre-existing conditions, mainly acute infectious illnesses in the period prior to IMD onset (n = 7) and previous medical problems that had been treated or resolved (n = 5). Septicaemia alone or with meningitis was the most common clinical presentation (72%) and more than a third reported complications after hospital discharge (37%), especially hearing loss (**Table 1**).

**Table 1:** Summary of key results of illness characteristics in children with laboratory confirmed invasive meningococcal disease in England.

Characteristic	Outcome Measure	% (n/N) or Median (IQR)
**Age**	<1y	33.0% (36/109)
	1-4y	45.0% (49/109)
	5-9y	15.6% (17/109)
	10-14y	5.5% (6/109)
	≥15y	0.9% (1/109)
**Clinical Presentation**	Septicaemia	34.7% (38/109)
	Meningitis	23.9% (26/109)
	Both	37.7% (40/109)
	Other/no answer	4.6% (5/109)
**Presenting symptoms**	Fever	87% (95/109)
	Headache	34% (37/109)
	Rash	77% (84/109)
	Neck stiffness	28% (31/109)
	Photophobia	28% (30/109)
	Vomiting	65% (71/109)
	Drowsiness/confusion	70% (76/109)
	Seizure	10% (11/109)
	Coma	5% (5/109)
	Other	43% (47/109)
**Median length of stay**	PICU (n = 53)	3 days (2 to 6)
	Total length of stay (n = 107)	6 days (4 to 8)
**Course of illness**	Time from onset to worst day (n = 106)	1 day (0 to 1)
	Time from worst day to follow up (n = 103)	133 days (90 to 153)
	Number of days ‘most unwell’ (n = 109)	4 days (2 to 4)
Complications*	Overall	36.7% (41/109)
	Hearing loss	20.2% (22/109)
	Fits/seizures	9.2% (10/109)
	Concentration/memory loss	5.5% (6/109)
	Amputations	2.8% (3/109)
	Other	15.6% (17/109)

*Figures total more than 41 as a single case may have more than one complication.

PICU = paediatric intensive care unit.

The VAS was well completed (99% for worst day of illness, 97% on day of completing questionnaire). The health index questions had lower completion rate of 60% (65/109) for the worst day and 68% (74/109) for the date of completion, with 55% (60/109) completing both. This was skewed towards older children: 59/73 (81%) completion rates for ≥1 year compared to 6/36 (17%) for <1 year-olds for the worst day of illness.

The health profile summary results for those who completed all five domains are shown in **Table 2**. The majority of responses reported the child being severely affected in all the five domains on the worst day of the disease. The median index score was -0.073 on the worst day of illness (IQR, -0.073 to 0.075) and 1.000 (IQR, 1.000 to 1.000, min value 0.186) on the date of completion of questionnaire.

**Table 2:** The EQ-5D health profiles responses for children with group B meningococcal disease in England. Numbers may not add to 100% due to rounding.

Domain		Worst day of illness (n = 65)		Day of completion (n = 74)	
		n	%	n	%
Mobility	No problems	**4**	6	**69**	93
	Some problems	**16**	25	**5**	7
	A lot of problems	**45**	69	**0**	0
Looking after myself	No problems	**5**	8	**68**	92
	Some problems	**12**	18	**5**	7
	A lot of problems	**48**	74	**1**	1
Doing usual activities	No problems	**4**	6	**67**	91
	Some problems	**2**	3	**5**	7
	A lot of problems	**49**	75	**2**	3
Having pain or discomfort	No problems	**3**	5	**64**	86
	Some problems	**12**	18	**9**	12
	A lot of problems	**50**	77	**1**	1
Feeling worried, sad or unhappy	Not worried, sad…	**5**	8	**69**	93
	A bit worried, sad…	**8**	12	**5**	7
	Very worried, sad…	**52**	80	**0**	0

Regression analysis demonstrated a high level of concurrence between the VAS and health index scores (adjusted r^2^ 0.917, standard error 0.138). After imputation, the median health index was -0.056 (IQR -0.073 to 0.102) on the worst day, and 1 (IQR 0.866 to1.000) on the date of completion of questionnaire.

**Fig 2:**
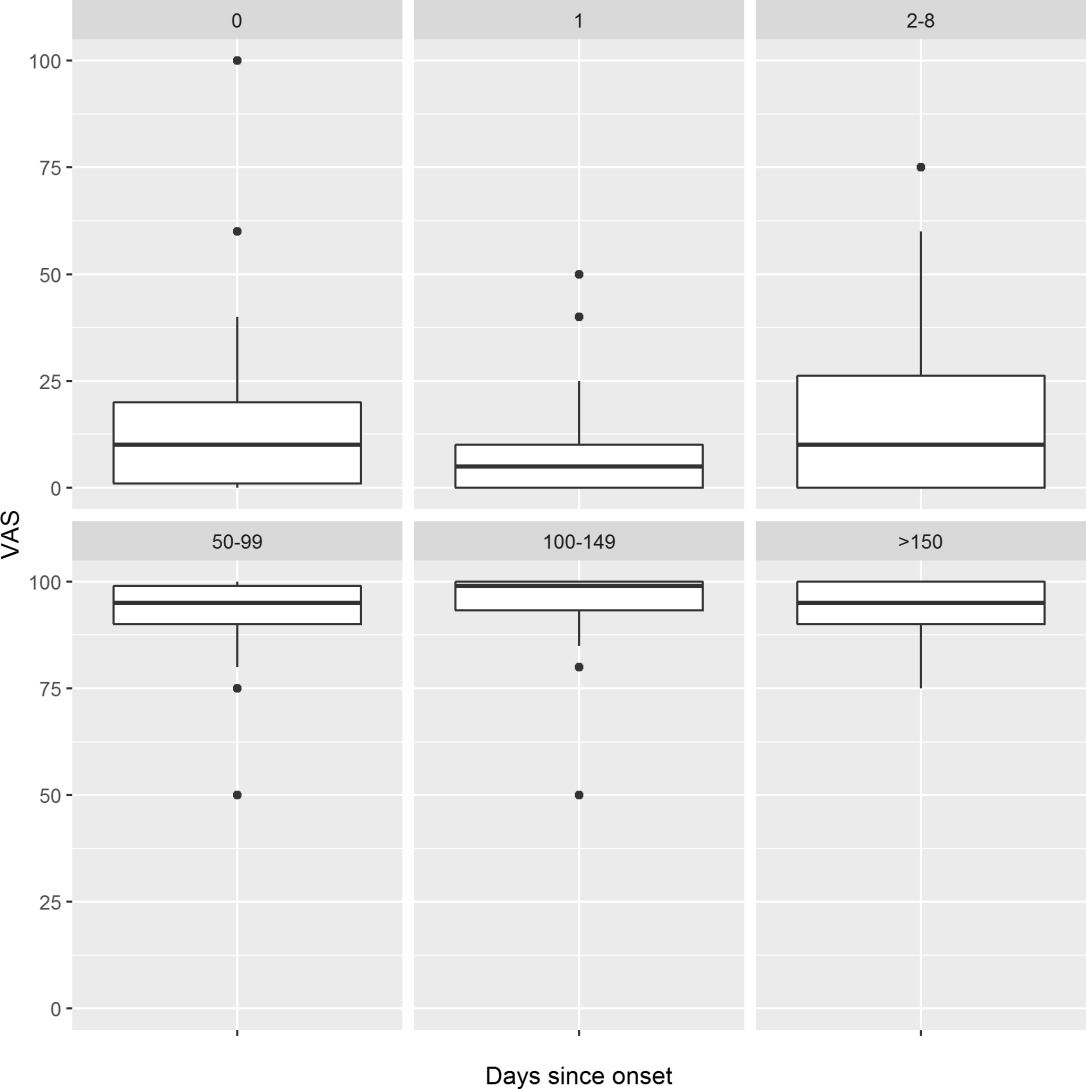
Box plot summary of EQ-5D VAS responses for children with laboratory-confirmed, group B meningococcal disease. The top row (0, 1, 2–8 days) are values recorded for the worst day, the bottom row (50–99, 100–149 and >150 days) for values recorded for day of completion of survey. The break points were choosen as to divide responses approximatley into thirds. The black dots represent individual data points that are outliers, defined as more than 1.5 times the interquartile range away from the median.

**Fig 3:**
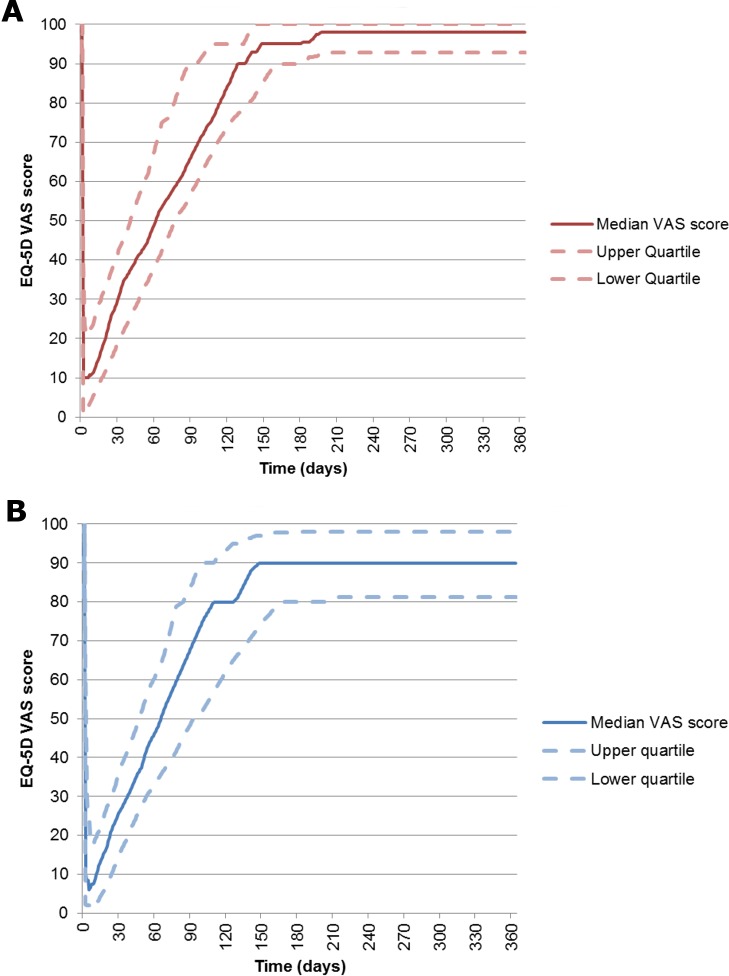
**Median EQ 5D VAS score for children with invasive group B meningococcal disease in England (a) without (n = 53) and (b) with long-term sequlae (n = 34).** The solid line represents the median score, while the dotted and dashed lines represent the upper and lower quartiles. ***The red line represents those without sequelae*, *the blue line those with sequelae***.

The median health state on the worst day, as described by VAS, was 6.5 (IQR, 0.0 to20.0) and, on the date of questionnaire completion, was 95.0 (IQR, 90.0 to 100.0) (**Fig 2**). When analysed separately, the health state of those cases with long-term sequelae (n = 41) was significantly worse at follow-up than those who recovered uneventfully (n = 65; 90.0 vs. 98.0; p<0.001), although there was no significant difference on the worst day of illness (5.0 v 10.0; p = 0.671) (**Fig 3**).

Fifty-three profiles were available using the collected health index data, and a total of 87 using the collected plus imputed data. Although not ‘true’ QoL measure (as it is not preference weighted) the VAS scores were also available for 87 profiles. The median numbers of Quality Adjusted Life Days lost for each of the three data sets, for each of the three scenarios in the sensitivity analysis (standard, maximal loss, minimal loss) are presented in Table 3. Within each scenario, the estimates from each dataset were similar.

**Table 3:** The median (interquartile) QALD lost, based on modelled health profiles, for children with laboratory-confirmed group B meningococcal disease.

All cases	Health index data	Health index data plus imputed results	*VAS*
n	53	87	*87*
Standard	73.8 (41.2–84.9)	66.7 (42.6–82.7)	*61*.*2 (39*.*9–76*.*5)*
Minimum loss	3.4 (2.1–5.4)	3.8 (2.1–5.5)	*3*.*6 (1*.*9–4*.*8)*
Maximum loss	142.0 (81.5–165.1)	129.5 (78.6–162.2)	*118*.*8 (75*.*8–148*.*6)*

There was a correlation between a lower VAS on the day of completion and longer length of hospital stay (r = -0.34, p<0.001) and with increasing number of nights in PICU (r = -0.67, p<0.001), although there was no significant difference in VAS for admission to PICU as a binary variable. There was also no significant association with clinical presentation, presenting symptoms, age (<1 year vs. ≥1 year) or accessing primary or community care prior to hospitalisation at either measured health state point.

## Discussion

This work has provided, for the first time, a quantitative estimate of QoL loss at the peak of illness and in the weeks to months following invasive MenB disease in children. Since the health profiles were extrapolated from a limited number of data points, they should not be used to predict recovery for individual children. Although the QoL value for the worst day of illness was completed retrospectively and, thus, potentially introducing recall bias, the magnitude of QoL loss is staggering, with the reported health state being at, or close to, the worst possible outcome imaginable. Reassuringly, though, almost all that loss is regained, with most individuals reaching close to perfect health within six months of the onset of illness. However 37% of individuals developed post-infectious sequelae, which had a significant impact on subsequent health state.

### Measuring health state in infants

A limitation of the study was the poor completion rate for the health index questions in infants (<1 year-olds), which prevented analysis of the full EQ-5D. This was, however, an expected event because many of the questions (for example, those relating to mobility or dressing) were irrelevant in this age-group. The EQ-5D is recommended by The National Institute for Health and Care Excellence (NICE) for adults, and, although the EQ-5DY has been adapted for children, it has not been validated for <7 year-olds and reference values for the health index are based on the general adult population (http://www.nice.org.uk/article/pmg20). Use of other tools such as the Canadian Health Utilities Index (HUI2 and HUI 3) or the Child Health Utility 9D tool, which have been designed specifically for use in children, may have resulted in a higher completion rate. However both have the same key limitations as EQ-5D –they are not validated for the youngest age groups and the weightings used to calculate health indices are based on adult preferences [12].

The substantially higher completion rate for the VAS component suggests that this simpler tool may be more applicable for younger age-groups. For parents of young children with IMD, marking the perceived health state on a linear scale from 0 to 100 is likely to be a more intuitive process.

As well as considering how to measure QoL in children, further research should focus on how significant acute, but temporary, loss should be weighted in the context of the person’s whole lifetime when attributing cost. There is also increasing evidence for subtle, but significant, long-term consequences following serious infection in early childhood [5,13]. For example, a significant proportion of apparently healthy adolescents who had meningitis in infancy have neurocognitive, educational and psychological problems [14]. The UK JCVI has previously noted that the EQ-5D methodology may be too insensitive to capture subtle changes, such as QALY loss for cognitive or hearing deficits [15]. This may result in an overestimation of the recovery in health status in the current study.

We did not assess the impact that the acute illness had on family members, but it is also of great and long lasting importance. These have been measured for meningitis by Al-Janabi and colleagues, who demonstrated, for example, that family members exposed to the after-effects of meningitis have 2.3 (95% CI: 1.8 to 2.9) times the odds of having depression or anxiety than those not exposed [13]. How best to weight these different aspects of the harm caused by the disease when considering the cost-effectiveness interventions is an area of uncertainty, and it may be appropriate to consider differential weightings for different age-groups.

Our findings provide important additional information on the course of MenB disease in surviving children, and the magnitude of the impact the illness has on the children. This information may be useful for policy setters when considering future control measures for meningococcal disease. This study has highlighted the inadequacies of currently available QoL assessment tools and raises the question as to how best measure the impacts of illness in young children, who often have the highest burden of potentially preventable infectious diseases.

## Supporting information

S1 Table Table summarising surveillance and questionnaire data for cases included in the final analysis. (PDF)
