# Supplementary material for: Short-term changes in the health state of children with group B meningococcal disease: A prospective, national cohort study
Source: PLoS One. 2017 May 18;12(5):e0177082. doi: 10.1371/journal.pone.0177082 (PMC5436659; doi:10.1371/journal.pone.0177082)
Supplement: S1 Table — (PDF) [file pone.0177082.s001.pdf]

idnumber	Age	Gender	VAS_worst_	VAS_now	QALY weigl	QALY weigl	QALY weigt
MBQ001	F	1-4	0	100	-0.073	-0.073	1
MBQ002	M	<1	0	100	-0.073	-0.073	1
MBQ003	F	<1	2	98		-0.089036	0.761
MBQ004	F	5-9	0	100	-0.073	-0.073	1
MBQ005	M	1-4	40	90	0.038	0.038	0.658
MBQ006	F	1-4	20	92	-0.073	-0.073	1
MBQ007	F	1-4	3	97	0.014	0.014	
MBQ008	F	<1	0	80		-0.110742	
MBQ009	M	<1	0	98	-0.073	-0.073	1
MBQ010	F	1-4	6	91	-0.073	-0.073	1
MBQ011	M	<1	5	90		-0.056478	
MBQ012	F	10-14	3	85	-0.073	-0.073	0.659
MBQ013	M	1-4	25	95	0.238	0.238	1
MBQ014	F	1-4	20	98	-0.073	-0.073	1
MBQ015	M	<1	100	100	0.761	0.761	0.761
MBQ016	F	1-4	5	100	-0.073	-0.073	0.752
MBQ017	M	5-9	11	91	-0.073	-0.073	0.761
MBQ018	M	<1	0	100		-0.110742	
MBQ019	F	<1	0	90		-0.110742	0.774
MBQ020	M	1-4	0	100	-0.073	-0.073	1
MBQ021	M	<1	5	100		-0.056478	
MBQ022	F	1-4	10	90	0.14	0.14	1
MBQ023	F	1-4	0	80		-0.110742	0.782
MBQ024	F	<1	30	90		0.2148434	
MBQ025	M	<1	10	95		-0.002213	
MBQ026	F	1-4	5	75	-0.073	-0.073	1
MBQ027	F	5-9	5	90	-0.073	-0.073	0.605
MBQ028	M	1-4	40	100	0.151	0.151	1
MBQ029	M	1-4	25	95	-0.073	-0.073	1
MBQ030	M	1-4	0	98	-0.021	-0.021	1
MBQ031	F	1-4	30	100	0.038	0.038	1
MBQ032	F	10-14	0	90	-0.012	-0.012	0.761
MBQ033	F	15-19	4	93	-0.073	-0.073	1
MBQ034	F	5-9	25	98	0.177	0.177	1
MBQ035	M	1-4	0	90	-0.073	-0.073	0.814
MBQ036	M	5-9	10	100	0.038	0.038	1
MBQ037	M	<1	5	95	-0.073	-0.073	
MBQ038	F	1-4	10	75		-0.002213	1
MBQ039	F	<1	30	100		0.2148434	1
MBQ040	F	<1	25	75		0.1605792	
MBQ041	F	<1	0	90	-0.073	-0.073	1
MBQ042	M	<1	1	100	-0.073	-0.073	
MBQ043	M	5-9	20	100	0.125	0.125	1
MBQ044	F	1-4	0	100	0.014	0.014	1
MBQ045	F	5-9	15	85	-0.073	-0.073	1
MBQ046	M	1-4	0	100	-0.073	-0.073	1
MBQ047	M	1-4	20	95		0.106315	1
MBQ048	M	5-9	5	100	0.075	0.075	1
MBQ049	M	5-9	15	100	-0.073	-0.073	1

MBQ050	F	1-4	2	98	-0.021	-0.021	1
MBQ051	F	10-14	20	85	0.177	0.177	0.751
MBQ052	M	1-4	75	97	0.186	0.186	0.761
MBQ053	M	1-4	0	100	0.125	0.125	1
MBQ054	F	<1	5	100		-0.056478	
MBQ055	M	5-9	3	93	-0.073	-0.073	1
MBQ056	M	<1	30	90		0.2148434	
MBQ057	M	5-9	14	98	-0.073	-0.073	1
MBQ058	M	<1	10	100		-0.002213	
MBQ059	F	10-14	0	100	1	1	1
MBQ060	M	<1	0	50		-0.110742	
MBQ061	M	<1	9	100		-0.013066	
MBQ062	F	5-9	5	96	-0.073	-0.073	1
MBQ063	F	1-4	0	100	-0.073	-0.073	1
MBQ064	F	1-4	5	95	-0.073	-0.073	1
MBQ065	F	1-4	7	80	0.014	0.014	0.186
MBQ066	F	1-4	10	100	0.066	0.066	0.752
MBQ067	F	<1	20	100		0.106315	
MBQ068	F	1-4	10	90	-0.073	-0.073	1
MBQ069	M	1-4	NA	90		0	
MBQ070	M	1-4	2	94	0.038	0.038	1
MBQ071	M	<1	10	100		-0.002213	
MBQ072	F	1-4	40	95	-0.073	-0.073	1
MBQ073	M	1-4	40	100		0.3233719	
MBQ074	M	1-4	23	98		0.1388735	
MBQ075	M	<1	0	95		-0.110742	0.761
MBQ076	M	1-4	5	95	1	1	1
MBQ077	M	<1	20	80		0.106315	
MBQ078	M	1-4	10	100	-0.073	-0.073	1
MBQ079	M	1-4	0	100	-0.073	-0.073	1
MBQ080	M	<1	5	95		-0.056478	
MBQ081	M	<1	0	95		-0.110742	
MBQ082	M	<1	0	100		-0.110742	
MBQ083	F	1-4	1	100		-0.099889	1
MBQ084	M	1-4	30	100	0.151	0.151	1
MBQ085	M	<1	60	100		0.5404287	
MBQ086	M	5-9	10	98	-0.073	-0.073	1
MBQ087	F	1-4	30	100		0.2148434	1
MBQ088	M	<1	NA	NA		0	
MBQ089	M	1-4	0	90		-0.110742	1
MBQ090	M	1-4	0	80		-0.110742	
MBQ091	M	<1	10	100		-0.002213	
MBQ092	F	<1	NA	NA		0	
MBQ093	F	1-4	0	75		-0.110742	
MBQ094	F	1-4	0	100	-0.012	-0.012	1
MBQ095	M	1-4	15	95	-0.021	-0.021	1
MBQ096	F	<1	0	90		-0.110742	
MBQ097	M	5-9	10	100	-0.073	-0.073	1
MBQ098	F	1-4	20	95		0.106315	
MBQ099	F	<1	0	100		-0.110742	
MBQ100	M	<1	10	95		-0.002213	
MBQ101	M	<1	15	90		0.0520508	

MBQ102	M	10-14	0	50	-0.073	-0.073	0.214
MBQ103	F	1-4	10	80		-0.002213	1
MBQ104	F	5-9	15	100	0.201	0.201	1
MBQ105	M	1-4	60	90	1	1	1
MBQ106	M	10-14	30	90	0.09	0.09	1
MBQ107	M	1-4	4	80		-0.06733	0.743
MBQ108	F	5-9	50	90	0.196	0.196	1
MBQ109	M	5-9	10	90	0.038	0.038	1

S1 Table. Table summarising surveillance and questionnaire data for cases included in the final

QALY weigl	days_onset	days_most_	days to foll	preexisting	hospital_ac	Number_of	icu/picu_ac	number_of
1.000	1	18	174	NO	YES	21	YES	7
1.000	1	5	194	YES	YES	8	YES	6
0.761	0	11	163	NO	YES	11	YES	6
1.000	0	5	146	NO	YES	6	YES	1
0.658	1	6	140	NO	YES	55	YES	13
1.000	0	2	80	NO	YES	5	YES	1
0.942	0	3	66	NO	YES	6	YES	1
0.757	1	8	202	NO	YES	10	YES	5
1.000	8	15	104	NO	NO		YES	1
1.000	0	2	169	NO	YES	3	NO	
0.866	0	4	190	NO	YES	6	YES	3
0.659	1	7	214	NO	YES	21	YES	4
1.000	0	2	203	NO	YES	1	NO	
1.000	0	14	NA	NO	YES	21	NO	
0.761	0	8	127	NO	YES	5	YES	5
0.752	1	3	188	NO	YES	3	NO	
0.761	361	6	194	YES	YES	9	NO	
0.975	0	7	119	NO	YES	11	YES	11
0.774	4	14	239	NO	NO		NO	
1.000	2	2	186	NO	YES	5	NO	
0.975	2	3	199	NO	YES	12	NO	
1.000	1	3	183	NO	YES	6	YES	6
0.782	7	4	208	NO	YES	3	NO	
0.866	0	3	134	NO	YES	5	NO	
0.920	NA	60	236	NO	YES	10	YES	4
1.000	1	2	207	NO	YES	3	NO	
0.605	0	8	176	NO	YES	12	YES	8
1.000	0	2	205	NO	YES	2	NO	
1.000	1	2	186	NO	YES	4	NO	
1.000	1	4	202	NO	YES	6	NO	
1.000	0	7	121	NO	YES	8	YES	5
0.761	1	4	207	NO	YES	5	NO	
1.000	1	2	94	NO	YES	3	NO	
1.000	0	3	156	NO	YES	2	NO	
0.814	2	4	159	NO	YES	7	NO	
1.000	0	4	144	NO	YES	7	YES	3
0.920	1	4	155	NO	YES	6	NO	
1.000	5	1	157	NO	YES	4	NO	
1.000	0	4	182	NO	YES	5	NO	
0.703	4	9	163	NO	YES	9	YES	2
1.000	1	4	178	NO	YES	7	NO	
0.975	0	3	152	NO	YES	7	YES	3
1.000	0	7	158	YES	YES	1	NO	
1.000	0	3	152	NO	YES	10	YES	5
1.000	0	2	150	NO	YES	3	NO	
1.000	1	14	185	NO	YES	27	YES	7
1.000	1	4	151	YES	YES	4	NO	
1.000	1	4	146	NO	YES	7	YES	4
1.000	0	1	171	YES	YES	5	YES	3

1.000	1	6	151	NO	YES	8	NO	
0.751	0	2	149	NO	YES	6	YES	2
0.761	NA	6	NA	NO	YES	4	NO	
1.000	0	4	144	NO	YES	3	NO	
0.975	0	5	136	NO	YES	7	NO	
1.000	2	12	143	NO	YES	8	NO	
0.866	3	5	143	NO	YES	5	NO	
1.000	1	5	142	NO	YES	6	YES	1
0.975	2	3	144	NO	YES	6	NO	
1.000	0	3	135	NO	YES	7	YES	2
0.432	0	7	131	NO	YES	26	YES	22
0.975	1	4	143	YES	YES	9	YES	2
1.000	1	4	139	NO	YES	10	YES	3
1.000	1	5	193	NO	YES	27	YES	
1.000	1	1	51	NO	YES	4	NO	
0.186	0	10	137	NO	YES	14	YES	12
0.752	0	2	111	NO	YES	2	NO	
0.975	0	2	111	NO	YES	8	NO	
1.000	1	4	151	NO	YES	7	YES	4
0.866	0	7	102	NO	YES	21	YES	2
1.000	0	2	151	NO	YES	5	YES	2
0.975	0	3	NA	NO	YES	7	YES	2
1.000	8	8	135	ACUTE	YES	5	NO	
0.975	1	1	96	NO	YES	2	NO	
0.953	2	1	93	NO	YES	1	NO	
0.761	1	11	122	NO	YES	28	YES	10
1.000	0	4	110	NO	YES	8	YES	1
0.757	3	3	93	NO	YES	7	NO	
1.000	2	4	126	NO	YES	7	YES	4
1.000	0	3	80	NO	YES	4	NO	
0.920	1	5	92	NO	YES	7	YES	7
0.920	1	5	86	NO	YES	6	NO	
0.975	1	3	81	ACUTE	YES	5	YES	2
1.000	0	2	80	NO	YES	4	YES	1
1.000	0	2	NA	YES	YES	2	NO	
0.975	2	9	110	NO	YES	7	NO	
1.000	0	2	75	NO	YES	6	NO	
1.000	2	2	138	ACUTE	YES	4	NO	
	1	2	74	NO	YES	2	NO	
1.000	2	3	NA	YES	YES	5	NO	
0.757	0	16	102	NO	YES	16	YES	9
0.975	3	2	70	NO	YES	4	YES	1
	1	3	66	NO	YES	6	YES	1
0.703	0	1	60	NO	YES	4	NO	
1.000	0	4	69	NO	YES	5	YES	2
1.000	0	2	58	NO	YES	1	NO	
0.866	1	9	68	NO	YES	14	YES	7
1.000	0	1.5	54.5	YES	YES	2	YES	1
0.920	0	2	101	NO	YES	2	NO	
0.975	7	NA	111	NO	YES	7	YES	7
0.920	0	5	97	NO	YES	6	YES	2
0.866	0	4	99	NO	YES	7	NO	

0.214	2	7	99	NO	YES	42	YES	19
1.000	0	4	106	ACUTE	YES	5	NO	2
1.000	1	5	77	NO	YES	4	NO	
1.000	0	1	70	NO	YES	9	YES	6
1.000	2	2	63	NO	YES	6	NO	
0.743	0	4	80	NO	YES	7	YES	3
1.000	1	NA	57	NO	YES	8	NO	
1.000	1	2	54	NO	YES	5	NO	

analysis

conditions_	conditions_	conditions_	hearing_los	conditions_	amputation	conditions_	excluded	excluded_r
NO	NO	NO		NO		NO		
NO	YES	MAYBE	Right	NO		NO	y	pre-existing
NO	NO	YES	Left	NO		NO		
NO	NO	NO		NO		NO		
NO	YES	YES	Left	NO		Left sided weakness to his arm, son		
NO	NO	NO		NO		NO		
NO	NO	NO		NO		YES movement in ®arm never had b		
NO	NO	NO		NO		YES still suffers sleepless nights & w		
NO	YES	YES	Left	YES	(fingersL, A	NO		
NO	NO	NO		NO		NO		
NO	NO	Waiting for tests		NO		NO		
YES	NO	NO		NO		YES She had a stroke pains in legs co		
NO	NO	NO		NO		NO		
NO	NO	NO		NO		YES Breathi	y	missing data
NO	NO	YES	Left	NO		NO		
YES	NO	NO		NO		Behaviour has got worse		
NO	NO	NO		NO		NO	y	pre-existing
NO	NO	NO		NO		Shakes/sick/not feeding/very sleepy		
NO	NO	YES	Left (mild)	NO		OTHER -Continuous none stopping		
NO	NO	NO		NO		NO		
NO	NO	NO		NO		NO		
NO	NO	NO		NO		NO		
NO	YES	NO		NO		NO		
NO	NO	NO		NO		NO		
NO	NO	NO		NO		NO	y	missing data
NO	NO	NO		NO		NO		
NO	NO	NO		NO		NO		
NO	NO	NO		NO		NO		
NO	NO	YES	Left	NO		NO		
NO	NO	NO		NO		NO		
NO	NO	NO		NO		NO		
NO	NO	YES	Right	NO		NO		
NO	NO	NO		NO		NO		
NO	NO	NO		NO		NO		
NO	NO	NO		NO		NO		
NO	NO	NO		NO		NO		
NO	NO	NO		NO		NO		
NO	NO	YES	Right	NO		NO		
NO	NO	NO		NO		NO		
NO	NO	YES	Right	NO		NO		
NO	NO	NO		NO		NO		
NO	NO	NO		NO		NO		
NO	NO	NO		NO		NO	y	pre-existing
NO	NO	NO		NO		NO		
NO	NO	NO		NO		Tiredness/Irritiable		
NO	NO	NO		NO		NO		
NO	NO	NO		NO		NO	y	pre-existing
NO	NO	NO		NO		NO		
NO	NO	NO		NO		NO	y	pre-existing

NO	NO	NO		NO		Unsteady when walking		
NO	NO	NO		NO		NO		
NO	NO	NO		NO		Ear infectio	y	missing dat
NO	NO	NO		NO		NO		
NO	NO	NO		NO		YES Temperature. Breathing Difficu		
NO	NO	NO		NO		NO		
NO	NO	NO		NO		NO		
NO	YES	YES		NO		NO		
NO	NO	NO		NO		NO		
NO	NO	NO		NO		NO		
NO	YES	Waiting for tests		YES	fingers-righ	NO		
NO	NO	NO		NO		NO	y	pre-existing
NO	NO	NO		NO		NO		
NO	NO	NO		NO		NO		
NO	NO	NO		NO		NO		
NO	NO	NO		YES	Fingers - bc	Skin grafts in various places and art		
NO	NO	NO		NO		NO		
NO	NO	NO		NO		NO		
NO	YES	YES	Right	NO		YES - Blood clot on brain		
NO	NO	NO		NO		NO	y	missing dat
NO	NO	NO		NO		NO		
NO	NO	NO		NO		NO	y	missing dat
NO	NO	NO		NO		NO	y	pre-existing
NO	NO	NO		NO		NO		
NO	NO	NO		NO		NO		
NO	NO	NO		NO		YES Toes curl over, has to have phy		
NO	NO	NO		NO		NO		
NO	NO	YES	Both	NO		NO		
NO	NO	NO		NO		YES no use of (L)arm, could not wal		
NO	NO	NO		NO		NO		
NO	NO	YES	Right	NO		NO		
NO	NO	NO		NO		NO		
NO	NO	NO		NO		Couldn't lift	y	pre-existing
YES	NO	NO		NO		NO		
NO	NO	NO		NO		NO	y	missing dat
NO	NO	NO		NO		NO		
NO	NO	NO		NO		NO		
NO	NO	Waiting for tests		NO		NO	y	pre-existing
NO	NO	NO		NO		NO	y	missing dat
NO	YES	YES	Both	NO		NO	y	missing dat
NO	NO	NO		NO		NO		
NO	NO	NO		NO		NO		
NO	NO	NO		NO		NO	y	missing dat
NO	NO	NO		NO		NO		
NO	NO	NO		NO		NO		
YES	NO	NO		NO		NO		
NO	YES	YES	Both	NO		NO		
NO	NO	NO		NO		NO	y	pre-existing
NO	NO	NO		NO		NO		
NO	NO	NO		NO		NO	y	missing dat
NO	NO	NO		NO		NO		
NO	NO	YES		NO		NO		

YES	NO	NO		NO		YES Kidney failure, Necrotic Lesions		
NO	NO	YES	(still being t	NO		NO	y	pre-existing
NO	NO	NO		NO		NO		
NO	NO	NO		NO		NO	n	
NO	NO	YES	Left	NO		NO	n	
YES	YES	NO		NO		NO	n	
NO	NO	NO		NO		YES could n	y	missing dat
NO	NO	NO		NO		NO	n	

eason

; condition

ne vision lost

efore, waiting to see Consultant
vill not be in a room on her own anymore

ouldn't walk or move very well

a

; condition
y/hard to wake
flu

a

; condition

; condition

; condition

a

lty and Body Stiffness

; condition

ificial skin on left wrist

a

a
; condition

sio

k or put weight on legs until last day in hospital

; condition

a

; condition
a
a

a

; condition

a

; on legs - regular appts/dressing changes with plastic surgeons 25/7/13
; condition

a
